# Overexpression of grape ABA receptor gene *VaPYL4* enhances tolerance to multiple abiotic stresses in *Arabidopsis*

**DOI:** 10.1186/s12870-022-03663-0

**Published:** 2022-06-02

**Authors:** Chong Ren, Yangfu Kuang, Yanping Lin, Yuchen Guo, Huayang Li, Peige Fan, Shaohua Li, Zhenchang Liang

**Affiliations:** 1 Beijing Key Laboratory of Grape Sciences and Enology, Key Laboratory of Plant Resource, Institute of Botany, Chinese Academy of Sciences, Nanxin Village 20, Xiangshan, Haidian District, Beijing, 100093 People’s Republic of China; 2 University of Chinese Academy of Sciences, Beijing, 100049 People’s Republic of China

**Keywords:** Abscisic acid, *Vitis amurensis*, *VaPYL4*, Overexpression, Abiotic stress

## Abstract

**Background:**

Abscisic acid (ABA) plays a crucial role in abiotic stress responses. The pyrabactin resistance (PYR)/PYR-like (PYL)/regulatory component of ABA receptor (RCAR) proteins that have been characterized as ABA receptors function as the core components in ABA signaling pathway. However, the functions of grape *PYL* genes in response to different abiotic stresses, particularly cold stress, remain less studied.

**Results:**

In this study, we investigated the expression profiles of grape *PYL* genes upon cold treatment and isolated the *VaPYL4* gene from *Vitis amurensis*, a cold-hardy grape species. Overexpression of *VaPYL4* gene in grape calli and *Arabidopsis* resulted in enhanced cold tolerance. Moreover, plant resistance to drought and salt stress was also improved by overexpressing *VaPYL4* in *Arabidopsis*. More importantly, we evaluated the contribution of *VaPYL4* to plant growth and development after the treatment with cold, salt and drought stress simultaneously. The transgenic plants showed higher survival rates, earlier flowering phenotype, and heavier fresh weight of seedlings and siliques when compared with wild-type plants. Physiological analyses showed that transgenic plants had much lower content of malondialdehyde (MDA) and higher peroxidase (POD) activity. Stress-responsive genes such as *RD29A* (*Responsive to desiccation 29A*), *COR15A* (*Cold responsive 15A*) and *KIN2* (*Kinase 2*) were also significantly up-regulated in *VaPYL4*-overexpressing *Arabidopsis* plants.

**Conclusions:**

Our results show that overexpression of *VaPYL4* could improve plant performance upon different abiotic stresses, which therefore provides a useful strategy for engineering future crops to deal with adverse environments.

**Supplementary Information:**

The online version contains supplementary material available at 10.1186/s12870-022-03663-0.

## Background

Plant growth and development are usually challenged by a variety of abiotic stresses such as cold, drought and salt stress. These abiotic stresses are big concerns for agriculture and could result in the loss of crop productivity [1]. After exposure to abiotic stress, plants need to coordinate physiological and biochemical processes and also gene expression to adapt to the severe environmental conditions [2]. Gene expression is generally associated with stress-induced hormone signaling, and phytohormone abscisic acid (ABA) has been reported to play a vital role in plant response to abiotic stress [3–6].

ABA signaling is initially triggered by ABA perception, which is accomplished by the binding of ABA receptors to ABA [7]. The pyrabactin resistance (PYR)/PYR-like (PYL)/regulatory component of ABA receptor (RCAR) that localizes to nucleus and cytosol is the predominant type of ABA receptors [7]. The PYR/PYL/RCAR (hereafter referred to as PYL) ABA receptor, together with protein phosphatase 2C (PP2C) and SNF1-related protein kinase 2 (SnRK2), has been reveled to form the core ABA signaling network, which is characterized as double-negative regulatory system [7, 8]. In the absence of ABA, PP2Cs bind and dephosphorylate SnRK2s, inhibiting the activities of SnRK2 proteins. When ABA molecules are recognized and bound by ABA receptors, the ABA-receptor complexes could physically interact with PP2Cs, resulting in the release of SnRK2s, which can activate the expression of downstream target genes [8–11]. In plants, many ABA receptors, protein phosphatases and kinases have been identified as ABA signaling components. In *Arabidopsis thaliana*, for example, there are 14 PYL members, 76 members of PP2C proteins and 10 SnRK2 protein kinases [12–14]. Following the study in *Arabidopsis*, the members of PYL, PP2C and SnRK2 family have also been isolated in other plants, such as rice [13, 15], maize [16–18], and tomato [19]. The ABA signal transduction pathway has been characterized in grapevine (*Vitis vinifera*) [20], and the members of grape PYL, PP2C and SnRK2 family are 9, 85 and 7, respectively [21].

As the stress phytohormone, ABA accumulation is rapidly increased in plants after exposure to abiotic stress, particularly drought and salinity [3, 8, 22]. The functions of ABA receptors in response to abiotic stress have been revealed in plants these years [23, 24]. Overexpression of *AtPYL4*/*RCAR10*, *AtPYL5*/*RCAR8* and *AtPYL13*/*RCAR10* enhanced drought resistance in transgenic *Arabidopsis* [25–27]. The *AtPYL9*/*RCAR1* was found to promote leaf senescence, which in turn increases drought resistance by limiting transpirational water loss and promoting water to flow to young tissues [27]. Moreover, *Arabidopsis* ABA receptor genes *AtPYL1*/*RCAR12* and *AtPYL3*/*RCAR13* were found to play a role in response to extreme temperatures, and their overexpressing plants showed increased tolerance to both cold and heat stress [28]. Ectopic overexpression of *OsPYL3* in *Arabidopsis* led to enhanced tolerance to drought and cold stress [29]. Similar results were also obtained when using the *OsPYL10* gene, whose overexpression resulted in improved tolerance to drought and cold stress in transgenic rice [30]. In 2012, VvPYL1 was first identified as an ABA receptor in grape [31]. Later, potential ABA receptors were systematically characterized in grape, and *VvRCAR7* was revealed to be induced by drought, salt and cold stress in leaves [32]. Interestingly, expression patterns of grape ABA receptors upon abiotic stress were different in leaves and roots. For instance, *VvRCAR5* expression was only induced in leaves by salt and cold [32]. However, the functions of most of grape *PYL* genes in response to abiotic stress remain largely unknown. Additionally, most of previous studies are focused on specific abiotic stress, and the joint influence of different stresses on plant growth is still less studied.

In this study, we explored the expression profiles of grape *PYL* family in response to cold stress in *V. amurensis*, and isolated the strongly induced *VaPYL4* gene to evaluate its contribution to cold resistance in grape and *Arabidopsis*. Furthermore, involvement of *VaPYL4* in drought and salt tolerance was also demonstrated in *Arabidopsis*. More importantly, we evaluated the contribution of *VaPYL4* to plant growth and development under multiple abiotic stresses (cold, salt and drought) conditions.

## Results

### *VaPYL4* is strongly induced by cold stress

**Fig. 1:**
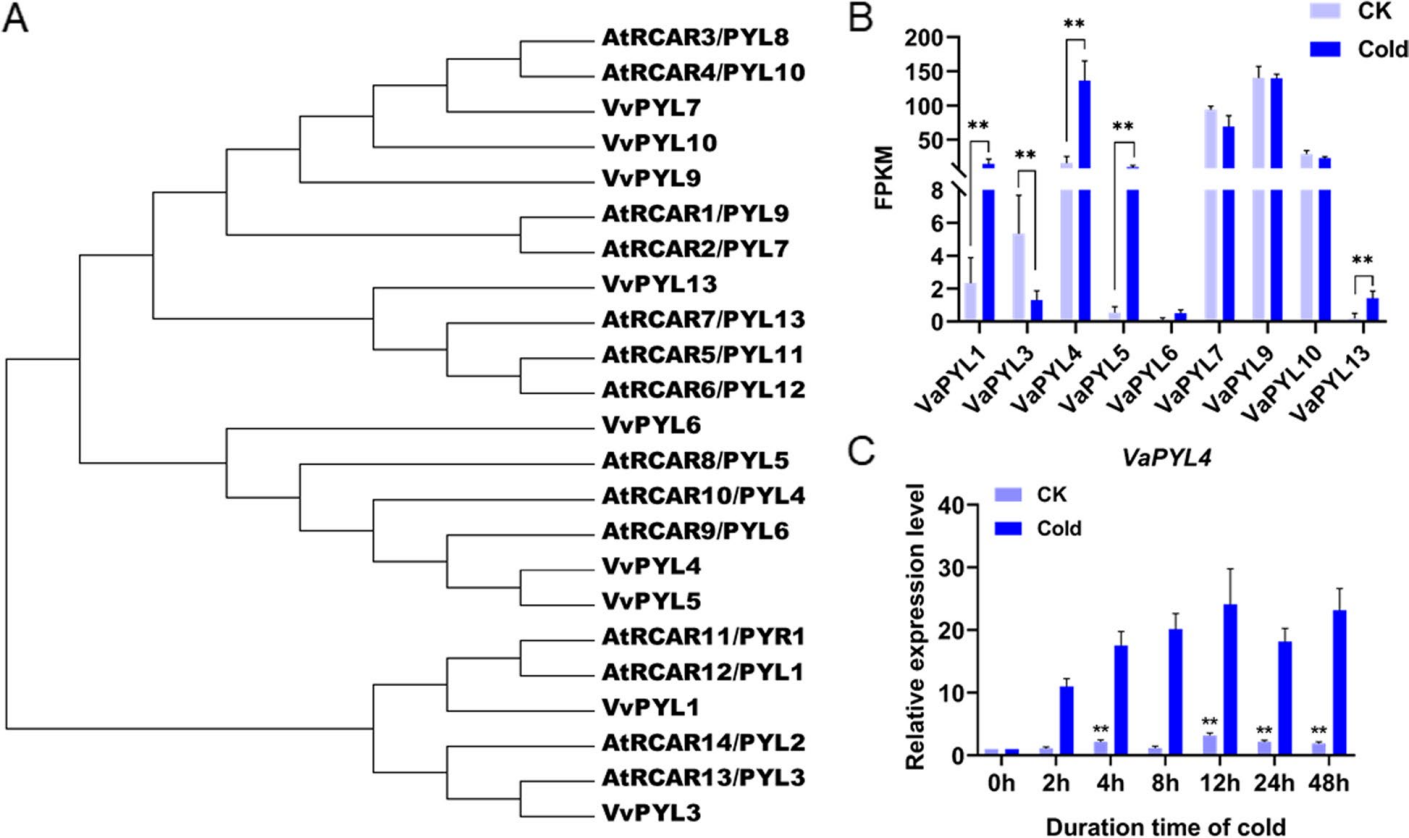
Expression of *VaPYL4* is induced by cold stress. **A** Phylogenetic analysis of PYL ABA receptors from *Vitis vinifera* and *Arabidopsis*. The phylogenetic tree was constructed using MEGA5.0 by NJ method with bootstrap replicates of 1000. **B** Expression profiles of *PYLs* in *V. amurensis* in response to cold treatment. The expression data were collected from the previous RNA sequencing results (GSE166247). Data are means of three replicates ± SD. **C** Expression of *VaPYL4* upon cold treatment. Two-month-old in vitro* V. amurensis* plants were treated at 4℃ for 48 h, and the leaves of cold-treated plants were collected at 0, 2, 4, 8, 12, 24 and 48 h during the cold treatment. The plants grown under normal conditions (25℃) were also sampled as the controls (CK) at each time point. The relative expression level of *VaPYL4* was determined by quantitative real-time PCR (qPCR). Data are shown as means ± SD from three biological replicates. Significant differences were determined using Student’s *t*-test. **P < 0.01

In an attempt to identify cold-responsive *PYL* genes in grapevine, we analyzed gene expression of the 9 members of *PYL* family (Fig. 1a) in *V. amurensis* based on our previous transcriptome data [33]. Among the 9 *PYL* genes, *VaPYL1*, *VaPYL4*, *VaPYL5* and *VaPYL13* were strongly induced by cold, whereas the expression of *VaPYL3* was significantly decreased in response to cold stress (Fig. 1b). Of the four up-regulated genes, *VaPYL4* exhibited the highest expression level upon cold stress (Fig. 1b). In addition, we investigated the expression profiles of *VaPYL4* in *V. amurensis* plants in response to cold stress. Two-month-old in vitro* V. amurensis* plants were treated at 4℃ for 48 h, and grapevine leaves were collected at 0, 2, 4, 8, 12, 24 and 48 h, respectively. The leaves from plants grown at room temperature (25℃) were also sampled at each time point as the controls. The expression of *VaPYL4* was detected using quantitative real-time PCR (qPCR). Interestingly, the *VaPYL4* gene exhibited changes in expression at 4, 12, 24 and 48 h without cold treatment (Fig. 1c), suggesting that the gene expression might be affected by circadian rhythms. However, the expression of *VaPYL4* was significantly increased under cold conditions (from 2 to 48 h) when compared with the corresponding controls (Fig. 1c). Based on these results, we thus selected *VaPYL4* as the candidate for further study.

### Overexpression of *VaPYL4* increases ABA sensitivity

**Fig. 2:**
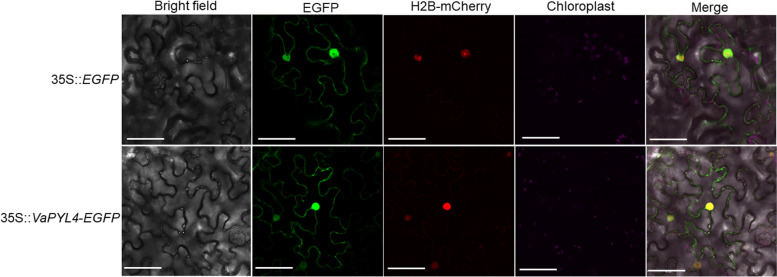
Subcellular localization of VaPYL4 protein**.** The coding sequence of *VaPYL4* was fused to the N-terminal of *EGFP*, which was driven by the CaMV 35S promoter. The EGFP fluorescence generated by the 35S::*VaPYL4*-*EGFP* construct in epidermal cells of *Nicotiana benthamiana* leaves was detected by using a confocal laser scanning microscopy. The histone H2B-mCherry was used as an indicator of nucleus. Scale bars correspond to 50 µm

Analysis of *PYL4* gene structure revealed that *PYL4* contains only one exon, which encodes a protein of 227 amino acids (Additional file 1: Figure S1). The coding sequence (CDS) of *PYL4* was amplified from *V. amurensis* and *V. vinifera* cv. Pinot Noir, respectively. Alignment of *VaPYL4* and *VvPYL4* showed that *VvPYL4* was totally identical with the reference sequence (‘Pinot Noir’ genome, PN40024), whereas *VaPYL4* contained 3 nucleotide changes (Additional file 1: Figure S2). However, analysis of corresponding amino acid sequences uncovered no difference between VaPYL4 and VvPYL4 (Additional file 1: Figure S2). It has been revealed that PYLs function as ABA receptors in nucleus and cytosol [7]. To analyze subcellular localization of VaPYL4, the CDS of *VaPYL4* was fused to the enhanced green fluorescent protein (EGFP) gene driven by the cauliflower mosaic virus (CaMV) 35S promoter (Additional file 1: Figure S2). The construct was infiltrated into *Nicotiana benthamiana* leaves for transient expression through *Agrobacterium*-mediated transformation. The results showed that the VaPYL4-EGFP fusion protein was localized to nucleus and cytosol (Fig. 2), which is consistent with previous results in *Arabidopsis* [7].

**Fig. 3:**
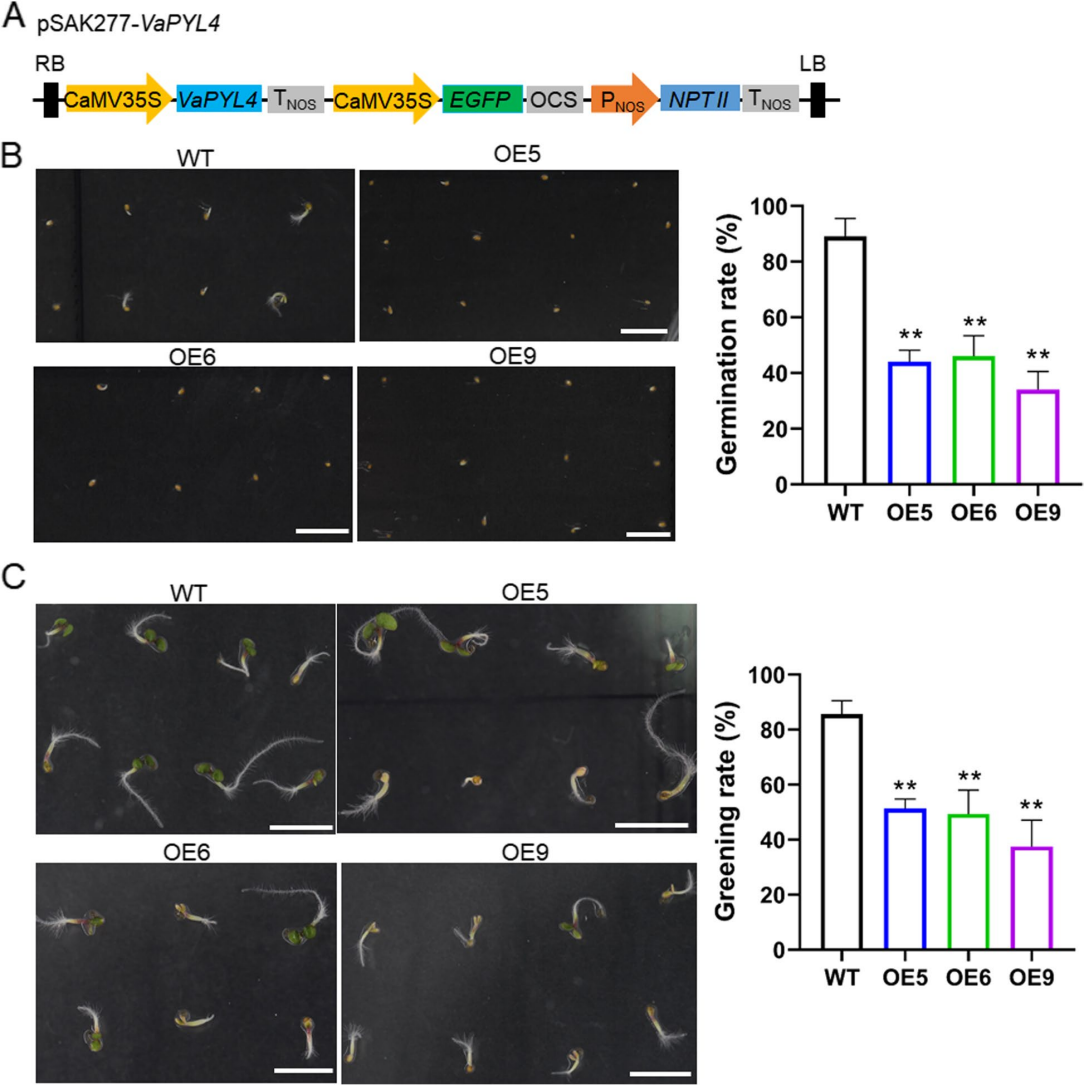
*VaPYL4*-overexpressing (OE) plants are hypersensitive to exogenous abscisic acid (ABA).** A** Schematic illustration of T-DNA region of the pSAK277-*VaPYL4* vector for gene overexpression. CaMV35S, cauliflower mosaic virus 35S promoter; T_NOS_, terminator of nopaline synthase gene; OCS, octopine synthase terminator; P_NOS_, promoter of nopaline synthase gene; RB, right border; LB, left border. **B** Seeds germination on 1/2 MS medium containing 0.3 µM ABA. After incubation at 4℃ for 2 d, wild-type (WT) and OE seeds were sown on 1/2 MS medium containing 0.3 µM ABA. The emergence of radicle was measured as germination, and the germination rates were calculated at 3 d after sowing. Scale bars correspond to 0.5 cm. **C** Cotyledon greening of WT and OE plants on 1/2 MS plates for 6 d. Scale bars correspond to 0.5 cm. Data are collected from three replicates and shown as averages of around 100 seeds. Date are presented as means ± SD. **P < 0.01

To test the function of *VaPYL4* in response to ABA, we cloned the CDS of *VaPYL4* into pSAK277 to develop the overexpression vector (Fig. 3a). The *VaPYL4*-overexpressing (OE) *Arabidopsis* plants (Additional file 1: Figure S3) were generated by using floral-dip method [34]. The seeds of two transgenic lines (OE5 and OE6) with similar expression level of *VaPYL4*, as well as the OE9 that exhibited a little higher *VaPYL4* expression, were used for germination test on 1/2 Murashige and Skoog (MS) medium supplemented with exogenous ABA. The results showed that there is no significant difference in germination rate between wild-type (WT) and transgenic lines in the absence of exogenous ABA (Additional file 1: Figure S4), which suggested that overexpressing *VaPYL4* in *Arabidopsis* had no much influence on seed germination under normal conditions. However, in the presence of ABA (0.3 μM), the seed germination of transgenic lines was obviously inhibited, exhibiting lower germination rates (40 ~ 44%) when compared with WT (> 87%) (Fig. 3b). Moreover, the inhibitory effect on seed germination was enhanced when ABA concentration was increased (Additional file 1: Figure S4). In addition, the average cotyledon greening rates of OE lines were also much lower than that of WT (Fig. 3c). For instance, the cotyledon greening rate of OE5 in the presence of 0.3 μM ABA was around 37.5%, whereas the greening rate of WT was over 85% (Fig. 3c). All these results suggested that *VaPYL4*-OE plants are hypersensitive to exogenous ABA.

### Overexpression of *VaPYL4* improves cold tolerance of grape callus and *Arabidopsis* plants

**Fig. 4:**
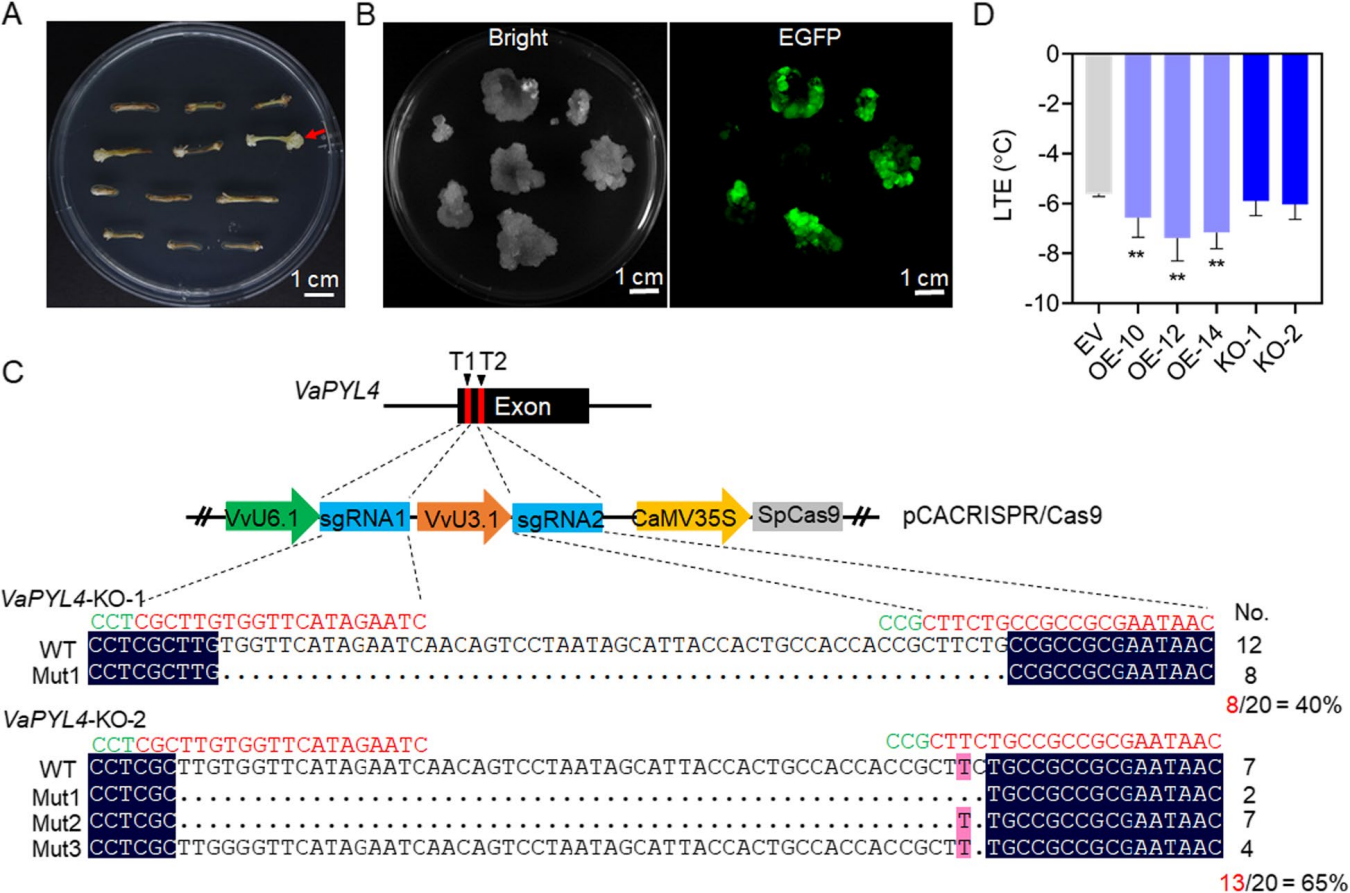
Overexpression of *VaPYL4* enhances cold tolerance of grape calli. **A** Grape calli induced from *V. amurensis* petioles on selection medium. The *V. amurensis* petioles were used as explants for co-culture with *Agrobacterium* cells that contain the *VaPYL4* overexpression vector. After co-culture, the *V. amurensis* petioles were placed on Gamborg medium supplemented with 50 mg/L kanamycin. The induced kanamycin-resistant callus is indicated by red arrow. **B** Detection of EGFP fluorescence in induced grape calli. **C** Knockout (KO) of *VaPYL4* in grape calli. Two sgRNAs targeting the exon of *VaPYL4* were cloned into the pCACRISPR/Cas9 vector. The results of targeted mutagenesis in *VaPYL4* in two independent calli (KO-1 and KO-2) were shown. The sequences of sgRNAs are shown in red, while the PAM (protospacer-adjacent motif) sequences are indicated in green. WT, wild-type sequence; Mut, mutated sequence. The number of analyzed amplicons and mutation efficiencies are shown on the right. **D** Values of low-temperature exotherms (LTEs) of transgenic grape calli. Data are shown as means ± SD collected from at least five biological replicates. **P < 0.01

As mentioned above, the expression of *VaPYL4* was induced by cold stress (Fig. 1). To investigate the function of *VaPYL4* in cold tolerance, we first developed *VaPYL4*-OE grape calli by introducing the pSAK277-*VaPYL4* construct (Fig. 3a) into *V. amurensis* petioles via *Agrobacterium*-mediated transformation. After kanamycin-dependent selection, the kanamycin-resistant calli were induced from petiole explants (Fig. 4a). The presence of *EGFP* reporter gene enabled us to screen transgenic calli rapidly according to the EGFP fluorescence (Fig. 4b). PCR identification was conducted by using *NPT II*-specific primers (Additional file 1: Table S1), and the results showed that all of the 15 tested grape calli contained exogenous T-DNA insertions (Additional file 1: Figure S5). Furthermore, the results of qPCR revealed that these calli exhibited increased expression levels (> tenfold) of *VaPYL4* compared with grape callus transformed with empty vector (EV) (Additional file 1: Figure S5). Three *VaPYL4*-OE grape calli lines (OE-10, OE-12 and OE-14) with high expression level were selected for subsequent analysis. In addition, we also developed the knockout materials of *VaPYL4* by using CRISPR/Cas9 (clustered regulatory interspaced short palindromic repeats/CRISPR-associated protein 9) technology. Two sgRNAs targeting the exon of *VaPYL4* were designed and ligated into pCACRISPR/Cas9 vector under the control of VvU6.1 and VvU3.1 promoter, respectively (Fig. 4c). After transformation and antibiotic-dependent selection, two independent calli lines were identified as *pyl4* mutants (Fig. 4c). Large fragment deletions (> 50 bp) were detected in the two knockout lines (KO-1 and KO-2), and the mutation efficiencies for KO-1 and KO-2 were 40% and 65%, respectively (Fig. 4c). Low temperature exotherms (LTEs) assay is usually used to evaluate cold tolerance of plant tissues or calli/cells [33, 35, 36]. Thus, the cold tolerance of KO-1 and KO-2, as well as the three OE lines, was evaluated by measuring LTEs using a differential thermal analysis system according to Sun et al. [36]. The LTEs of EV, OE-10 and KO-2, for example, were measured as -5.61, -6.57 and -6.04℃, respectively (Additional file 1: Figure S5). The OE lines exhibited significantly lower LTEs when compared with EV, while the KO lines, however, showed no obvious alterations in LTEs as expected (Fig. 4d). The possible reason is that the presence of wild-type cells in the KO lines might affect the measurement of LTEs. Moreover, multiple *PYL* genes may function redundantly in cold response, considering that *VaPYL1*, *VaPYL5* and *VaPYL13* were also induced by cold treatment (Fig. 1b). These results showed that overexpressing *VaPYL4* could enhance cold tolerance of grape calli.

**Fig. 5:**
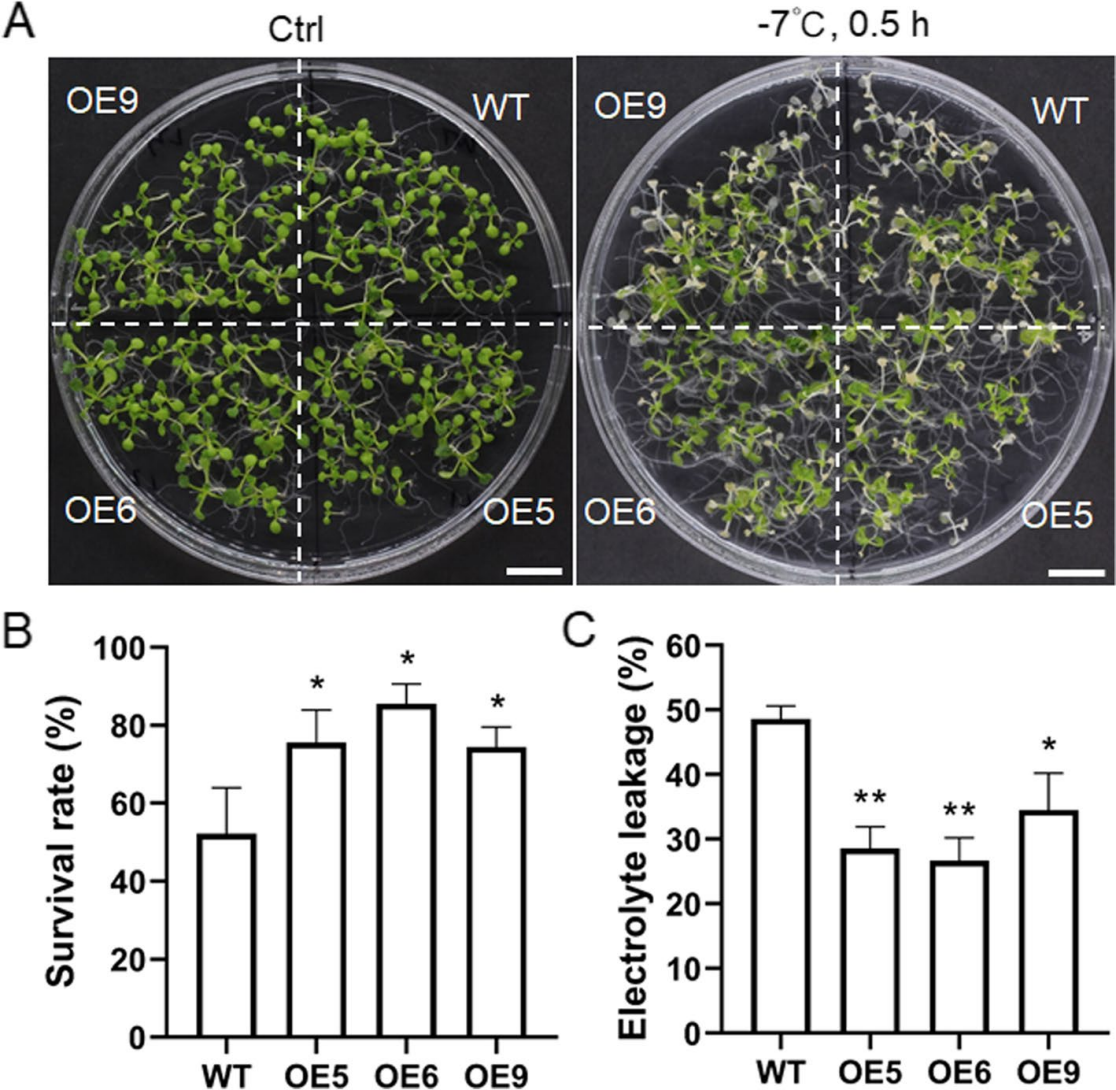
Overexpression of *VaPYL4* enhances cold tolerance of transgenic *Arabidopsis* plants. **A** Freezing phenotypes of WT and OE plants. WT and OE plants were grown on 1/2 MS medium at 22℃ for 10 days before being subjected to freezing treatment. The plates were kept at -7℃ for 0.5 h and then incubated at 4℃ for 12 h. Representative photos were taken after recovery for 3 days under normal conditions. Scale bars correspond to 1 cm. **B** Survival rates of *Arabidopsis* plants after freezing treatment. **C** Electrolyte leakage of *Arabidopsis* plants after freezing treatment. Data are means ± SD. *P < 0.05; **P < 0.01. Similar results were observed in at least three independent experiments

Freezing treatment was also performed with *VaPYL4*-OE *Arabidopsis* plants. Both WT and OE lines were treated at -7℃ for 0.5 h, and the survival rates of OE5, OE6 and OE9 were obviously higher than that of WT (Fig. 5a-b). Moreover, the electrolyte leakage of OE plants was much lower than that of WT after freezing treatment (Fig. 5c). These results showed that ectopic overexpression of *VaPYL4* in *Arabidopsis* could enhance its tolerance to freezing stress. It seems that OE5 and OE6 outperformed OE9 during the freezing treatment (Fig. 5), and the two lines were therefore chosen for subsequent treatments.

### Overexpression of *VaPYL4* enhances the tolerance of *Arabidopsis* to salt and drought stress

**Fig. 6:**
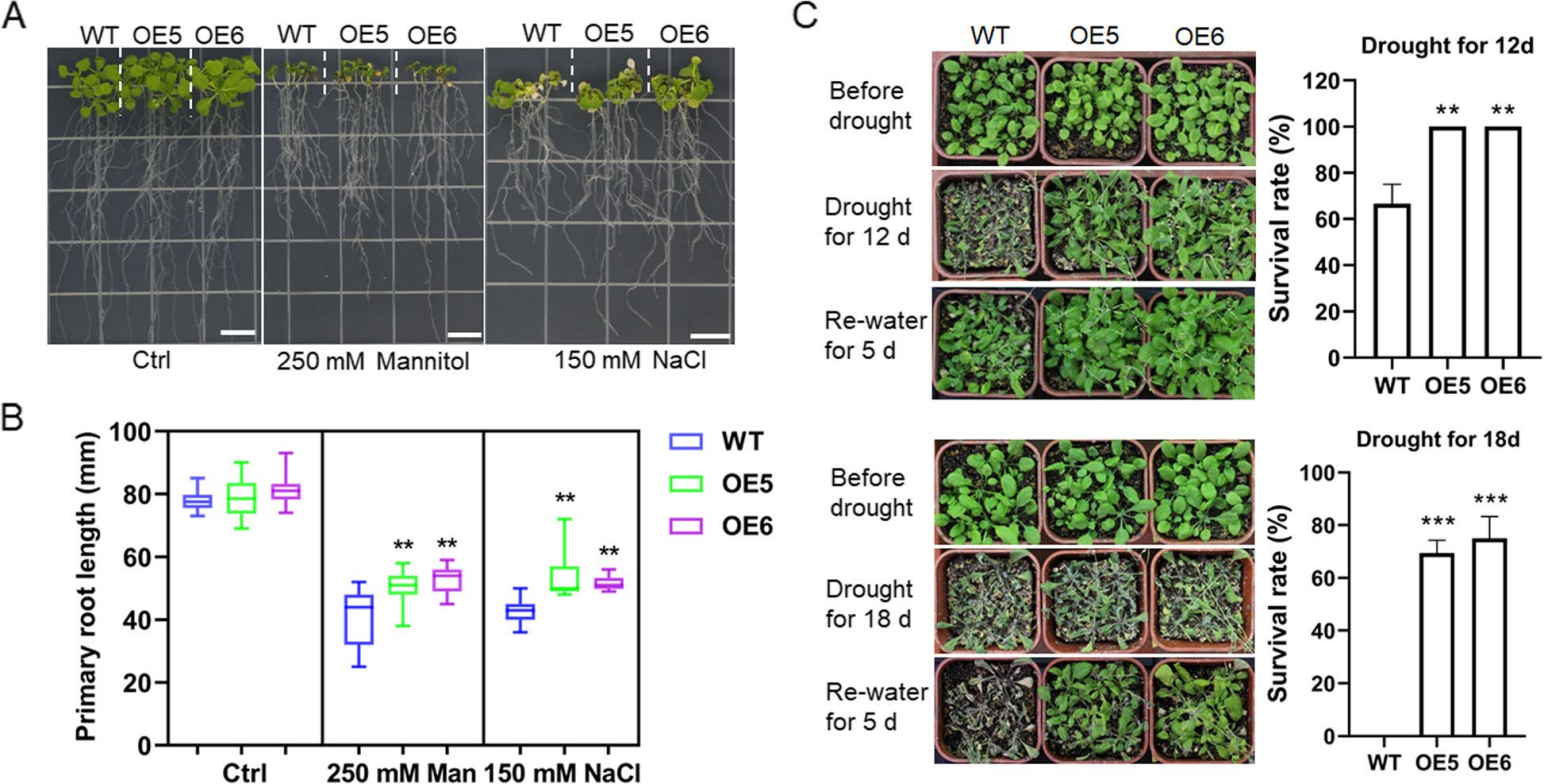
Overexpression of *VaPYL4* improves resistance of transgenic *Arabidopsis* plants to salt and drought stress. **A** Phenotypes of WT and OE plants under salt (NaCl) and mannitol conditions. Plants growing on normal 1/2 MS medium were shown as the control (ctrl). Scale bars: 1 cm. **B** Length of primary roots of *Arabidopsis* plants shown in (**A**). At least 25 seedlings were used for the measurement for each genotype. **C** Phenotypes of WT and OE plants under drought conditions. Drought treatment was performed by withholding water for 12 or 15 days, then the plants were re-watered for 5 days. Survival rates were measured after the recovery. Data are means ± SD. **P < 0.01; ***P < 0.001. Similar results were observed in three independent experiments

To investigate whether *VaPYL4* participates in the response to other abiotic stresses such as salt and drought, we performed salt and drought treatment, respectively, with OE5 and OE6 plants. One-week-old *Arabidopsis* plants were transferred onto the 1/2 MS medium supplemented with 250 mM mannitol or 150 mM NaCl for treatment. The plant growth of WT and OE lines was obviously inhibited by the presence of mannitol (Fig. 6a), which was used to mimic osmotic or drought stress. Similar phenotypes were also observed for the plants under salt treatment (Fig. 6a). The primary root length of *Arabidopsis* plants was obviously decreased under stress conditions (Fig. 6b). However, compared with WT, the two transgenic lines, OE5 and OE6, had longer primary roots (Fig. 6b), which suggested that OE5 and OE6 were more resistant to drought and salt stress. Moreover, drought resistance of OE5 and OE6 was further evaluated using pot experiments. The two OE lines were subjected to drought stress and re-watering, and the results showed that the two OE lines were more resistant to drought than WT (Fig. 6c). Compared with an ~ 66.7% survival rate of WT plants, up to 100% of OE5 and OE6 plants survived from a 12-d drought stress treatment followed by a 5-d recovery period. Intriguingly, no WT plants survived after an 18-d drought stress treatment followed by a 5-d recovery period. By contrast, over 69% of OE5 and OE6 plants survived from the treatment (Fig. 6c). All these results showed that overexpression of *VaPYL4* enhanced the tolerance of transgenic *Arabidopsis* to salt and drought stress.

### Overexpression of *VaPYL4* reduces adverse effect on plant growth caused by multiple abiotic stresses

**Fig. 7:**
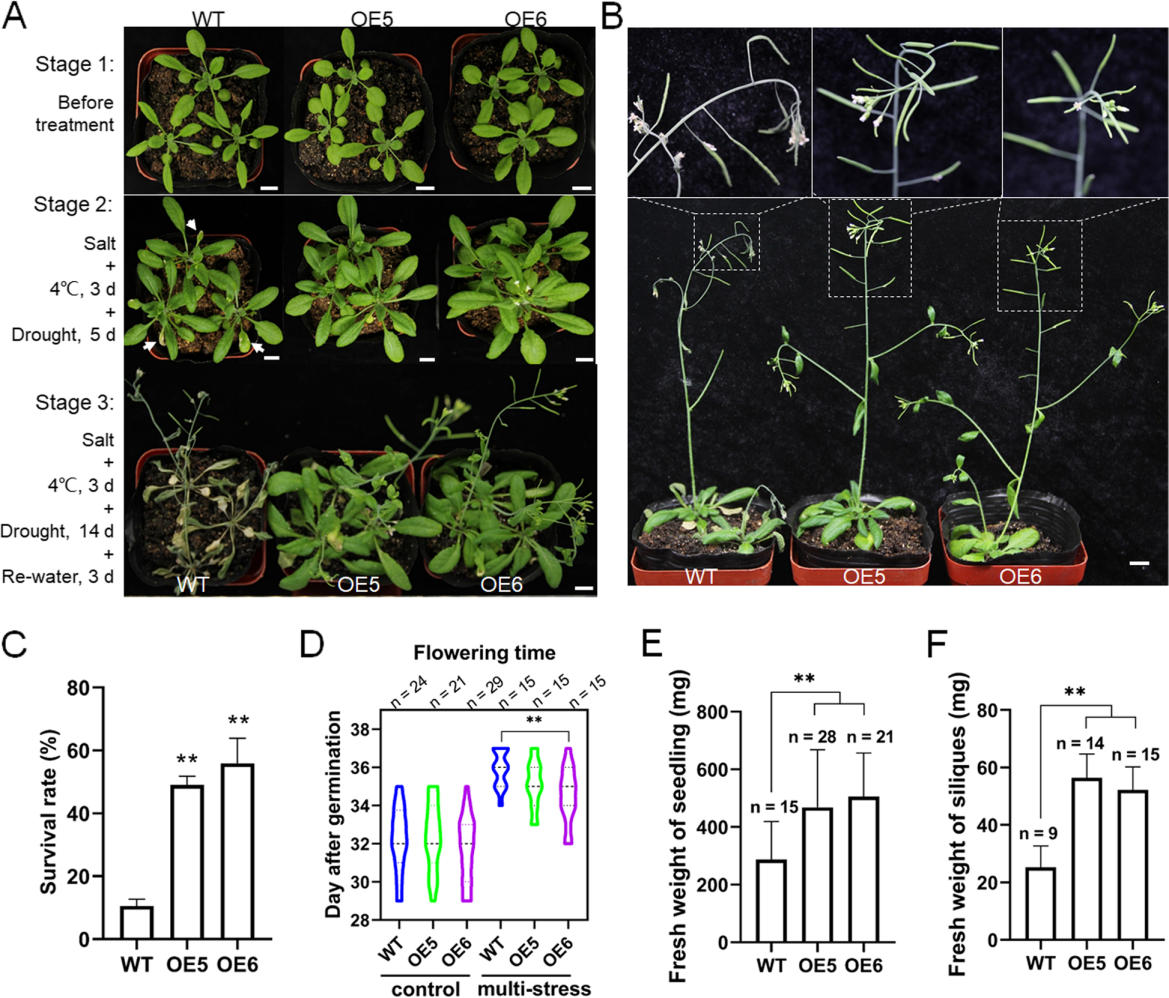
Overexpression of *VaPYL4* improves plant performance after multi-stress treatment. **A** Phenotypes of WT and OE plants during the treatment with different abiotic stresses. Three-week-old seedlings were first watered with 100 mM NaCl solution for 5 days and then treated at 4℃ for 3 days, followed by a drought treatment by withholding water for 14 days and a 3-day recovery period. Representative images were taken before treatment (stage 1), at 5 days (stage 2) of drought treatment, and at the end of the treatment (stage 3), respectively. The damages observed in WT leaves at stage 2 were indicated by white arrows. Scale bars: 1 cm. **B** Images showing the development of seedlings and siliques after multi-stress treatment. Photographs were taken at 50 days post-germination. Scale bar: 1 cm. **C** Survival rates of WT and OE plants. Data are collected from three replicates, and each replicate consists of at least 45 seedlings. **D** Flowering time of WT and OE plants treated with or without abiotic stresses. The plants with at least one flower were considered to be at the flowering stage. Data are collected from three replicates, and each replicate consists of at least 10 and 50 seedlings for control and multi-stress treatment, respectively. The number of plants survived from treatment and used for flowering time record is shown above the figure. **E**–**F** Fresh weight of seedling (**E**) and siliques (**F**). The individual plants without roots were used for the measurement of fresh weight (FW). For the measurement of FW of siliques, all the siliques from the same genotype were pooled and a number of 10 siliques were collected as a sample. The replicates from three experiments are shown above the bars. Data are means ± SD. **P < 0.01

In the experiments described above, we evaluated the tolerance of *VaPYL4*-OE plants to cold, salt and drought stress separately. Nevertheless, as sessile organisms, plants are usually threatened by multiple stresses simultaneously. Based on the results we have obtained, we speculated that overexpressing *VaPYL4* may also help to mitigate adverse effect on growth of transgenic plants under conditions of multiple abiotic stresses. To test this hypothesis, 3-week-old seedlings of WT, OE5 and OE6 were first irrigated with 100 mM NaCl solution, and 5 days later the pot seedlings were treated at 4℃ for 3 d, followed by a 14-d drought treatment and 3-d recovery period. The treatment was divided into three different stages (stage 1–3) as shown in Fig. 7a. Stress-induced damages were observed in WT leaves at stage 2 during the treatment (Fig. 7a). Most (about 90%) of WT seedlings were dead at stage 3, while ~ 49% of OE5 and ~ 55.9% of OE6 seedlings successfully survived from the successive treatments of different abiotic stresses (Fig. 7a, c). Moreover, development of the seedlings was also attenuated by the treatment with multiple stresses when compared with those plants grown under normal conditions (Fig. 7b; Additional file 1: Figure S6). However, the growth of OE5 and OE6 seedlings was less affected by multi-stress treatment (Fig. 7b). Though all the seedlings showed a delayed flowering phenotype when treated with abiotic stresses (Fig. 7d), most (33–53%) of transgenic seedlings exhibited an earlier (1–2 d) flowering phenotype when compared with WT plants (Fig. 7d). More importantly, analysis of the fresh weight (FW) of individual seedlings showed that the FW of transgenic plants outweighed the controls (Fig. 7e). In addition, similar results were also observed in the measurement of FW of siliques (Fig. 7f). These results suggested that the OE5 and OE6 plants had more biomass than WT plants after multi-stress treatment.

**Fig. 8:**
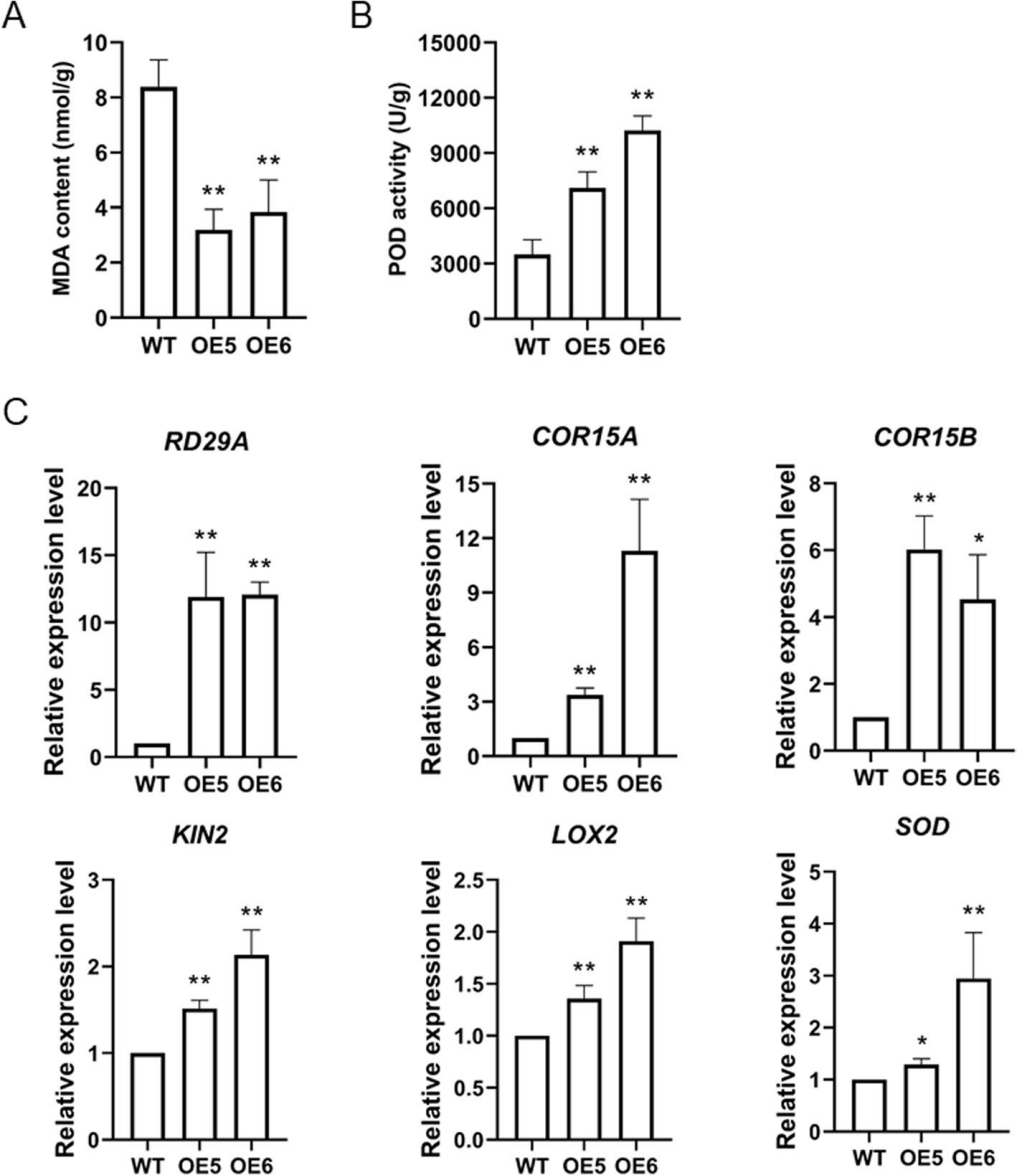
Determination of MDA (malondialdehyde) content, POD (peroxidase) activity and expression of stress-responsive genes. **A** MDA content measured in WT and OE plants during the multi-stress treatment. **B** POD activity detected in WT and OE plants during the multi-stress treatment. **C** Expression of stress-responsive genes in WT and OE plants. The expression levels of stress-responsive genes in OE plants relative to WT were determined by qPCR. Data are mean values ± SD of three biological replicates. *P < 0.05; **P < 0.01

Physiological changes were investigated at stage 3 during the treatment. The malondialdehyde (MDA) contents were significantly lower whereas the peroxidase (POD) activities were much higher in OE5 and OE6 plants (Fig. 8a, b). Investigation of expression profiles of stress-responsive genes revealed that the expression levels of *RD29A* (*Responsive to desiccation 29A*), *COR15A* (*Cold responsive 15A*), *COR15B* and *KIN2* (*Kinase 2*) in OE5 and OE6 were much higher relative to WT (Fig. 8c). Furthermore, the jasmonic acid (JA) biosynthetic related gene *LOX2* (*Lipoxygenase 2*) and the superoxide gene *SOD* [37] were also up-regulated in OE5 and OE6 plants (Fig. 8c).

The seedlings at the flowering stage were also used for the experiment. The results showed that *Arabidopsis* seedlings at the flowering stage were more sensitive to stress treatment, and unsurprisingly, the OE5 and OE6 plants still outperformed the WT plants (Additional file 1: Figure S7). Taken together, our results showed that overexpression of *VaPYL4* could help *Arabidopsis* plants to survive from severe environment conditions and mitigate adverse effect provoked by different abiotic stresses on plant growth.

## Discussion

ABA content was generally increased in plants upon abiotic stresses such as drought and salinity [8, 38–40], which greatly affect plant growth and distribution [41, 42]. Most of previous studies on ABA-dependent signaling were carried out with a focus on plant response to drought or high salinity [43–47]. Cold tolerance involved in ABA signaling is relatively less studied. Recently, overexpression of *PYL* genes from *Arabidopsis* and rice was found to enhance cold tolerance of transgenic plants [28–30]. In grapevine, several ABA receptor genes, including *VvRCAR5*/*PYL4* and *VvRCAR7*, were found to be induced by cold stress [28]. Consistent with this result, the *VaPYL4* gene responded to cold treatment as well (Fig. 1). Moreover, the function of *VaPYL4* in cold tolerance was further confirmed by gene overexpression in both grape calli and *Arabidopsis* plants (Fig. 4, 5). These results provide evidence for ABA-mediated cold tolerance in grapevine.

In addition to cold tolerance, drought and salt resistance was also improved in transgenic *Arabidopsis* plants (Fig. 6), indicating that the *VaPYL4* gene has a great potential for broader applications. A recent study reported that overexpression of a wheat ABA receptor increased water-use efficiency and improved grain production under drought condition [48]. The *VaPYL4* gene reported here can also serve as a promising candidate for grape and crops improvement. In natural environment, plants need to adapt to different biotic and abiotic stresses. Nevertheless, current studies usually focus on individual abiotic stress. In the present study, we treated the *VaPYL4*-OE plants with salt, cold and drought stress, and found that the OE5 and OE6 seedlings were less affected by these treatments when compared with WT plants, showing an earlier flowering phenotype, higher survival rate, and heavier weight of seedlings and siliques (Fig. 7). Measurement of MDA content, which is an indicator of plasma membrane damage, showed that OE5 and OE6 plants had much lower level of MDA (Fig. 8a). On the contrary, the activity of POD that helps to scavenge reactive oxygen species generated by abiotic stresses was higher in OE5 and OE6 (Fig. 8b). Regulation of stress-related gene expression is an important mechanism employed by plants to cope with abiotic stresses [2]. Notably, regulatory pathways triggered by different stresses may share the common targets. The *COR15A* and *RD29A* gene could be induced by both drought and cold stress [49]. We investigated the expression of stress-induced genes and found that all these famous abiotic stress-responsive genes, namely *RD29A*, *COR15A*, *COR15B* and *KIN2*, were significantly up-regulated after multi-stress treatment (Fig. 8c). Moreover, the transcripts of the two genes *LOX2* and *SOD*, which had been reported to be involved in drought resistance [37], were also highly enriched in OE5 and OE6 seedlings (Fig. 8c). All these results suggest that overexpression of *VaPYL4* improves plant performance upon different abiotic stresses.

We adopted the 35S promoter for overexpression of *VaPYL4* in *Arabidopsis* (Fig. 3a). Though constitutive expression of *VaPYL4* had no much influence on plant growth and development under normal conditions (Fig. 7a; Additional file 1: Figure S6), stress-inducible promoters like *RD29A* and tissue-specific promoters from plant genes could be used instead of 35S promoter during genetic improvement of grapevine and other crops in the future. Recently, the CRISPR/Cas9 has been used to improve the crop traits by editing the regulatory elements/promoters of genes of interest [50, 51]. Elevated ABA receptor expression could be achieved through promoter editing by using CRISPR/Cas9 technology. More importantly, the CRISPR reagents could be delivered into plant cells in ribonucleoprotein complex or by transient expression [52], which provides an alternative to generating transgene-free edited plants.

## Conclusions

In summary, the present study revealed that the grape ABA receptor gene *VaPYL4* plays an important role in plant response to cold, drought and salt stress, and overexpression of *VaPYL4* could improve plant performance upon exposure to these stresses simultaneously. Importantly, our study provides a useful strategy for engineering future crops to deal with increasingly adverse environments.

## Materials and methods

### Plant materials, culture conditions and treatments

The seedlings of *V. amurensis* and *V. vinifera* cv. Pinot Noir were grown in germplasm resources orchard at Institute of Botany, Chinese Academy of Sciences, Beijing, China. The derived in vitro plants of *V. amurensis* and *V. vinifera* were cultured in an artificial climate chamber with a 16-h light/8-h dark photoperiod at 25 ± 1℃. Two-month-old *V. amurensis* plants were used for cold treatment, which was conducted as previously described with some modifications [33]. The grapevine plants with uniform growth status were divided into two groups: one group of plants were transferred to the incubator set to 4℃ for cold treatment and the other kept at 25℃ were used as the control. The leaves from three different plants were collected as a biological replicate, and three replicates were prepared for each time point (0, 2, 4, 8, 12, 24 or 48 h).

*Arabidopsis thaliana* ecotype Columbia 0, which was kindly provided by professor Haiping Xin (Wuhan Botanical Garden, Chinese Academy of Sciences), was used as wild type. For ABA treatment, *Arabidopsis* seeds were surface-sterilized in10% (v/v) bleach for 15 min and rinsed five times with sterilized water. After a short culture at 4℃ for 2 d in the dark, the *Arabidopsis* seeds were grown on 1/2 MS medium (pH 5.8) supplemented with 0.3% phytagel, 1.5% sucrose and different concentrations of ABA (0, 0.3, 0.5 or 1.0 μM) under light conditions. The rates of germination, which was characterized by the obvious emergence of radicle through the seed coat, were calculated at 3 d (0.3 μM ABA) or 7 d (0.5 or 1.0 μM ABA) after sowing. The percentage of plants with green expanded cotyledons in the presence of 0.3 μM ABA was scored at 6 d after sowing. A number of 20 seeds were placed evenly on one plate for each genotype. Five plates were regarded as five replicates for the germination test. For freezing treatment, surface-sterilized seeds germinated and grew on 1/2 MS plates for 15 d. Then the temperature dropped by 1℃ per minute until reaching to -7℃, and the plants were kept at -7℃ for 0.5 h. After treatment, the plants were incubated at 4℃ for 12 h and then cultured under normal conditions for 3 d. The freezing treatment was conducted in the dark, and the survival rates and ion leakage were measured after plant recovery. For salt and mannitol treatment, 7-d-old plants were transferred to 1/2 MS medium with 150 mM NaCl or 250 mM mannitol, and the plants were cultured vertically for another 12 d. The length of primary roots was then recorded. All the plates were incubated in an incubator at 22℃ under a 16-h light/8-h dark photoperiod.

For drought treatment, 2-week-old plants grown on 1/2 MS medium were transplanted to soil in pots (7 cm in diameter and 7 cm in depth), and twelve seedlings were planted in each pot. *Arabidopsis* plants were cultured for 14 d in a growth room at 24 ± 1℃ under a long day condition (16-h light/8-h dark) with a light intensity of 100 μmol m^−2^ s^−1^. Drought treatment was conducted by withholding water for 12 d or 18 d. Drought recovery and survival rates were observed 5 days after re-watering.

For the multi-stress treatment at the seedling stage, *Arabidopsis* seeds were sown in the soil, and 3-week-old plants were irrigated with 100 mM NaCl solution for five days. Then the pots were transferred to a prechilled incubator set to 4℃ and kept at the temperature for 3 d. After cold treatment, the plants were subjected to drought treatment by withholding water for 14 d under normal conditions. Flowering time was recorded during the treatment, while survival rates and fresh weight of seedlings and siliques were measured at the end of treatment.

### Gene cloning and subcellular localization

The leaves of *V. amurensis* and *V. vinifera* plants were used to prepare total RNA, which was then adopted for cDNA synthesis by using the HiScript III 1st Strand cDNA Synthesis Kit (Vazyme) following the manufacturer’s instruction. The full-length CDS of *PYL4* was amplified from the prepared cDNA libraries of *V. amurensis* and *V. vinifera*, respectively, by PCR with the primers PYL4-PCR-F and PYL4-PCR-R (Additional file 1: Table S1) using the KOD-Plus-Neo Kit (TOYOBO). The amplified fragments were cloned into the pLB cloning vector (TIANGEN) for Sanger sequencing assay.

To generate the expression vector for subcellular localization, the verified sequence of *VaPYL4* without stop codon was amplified from the pLB vector using the primers PYL4-2300-F and PYL4-2300-R and ligated into the modified pCAMBIA2300-EGFP vector through *Bam*HI site via homologous recombination (HR) by using the ClonExpress II One Step Cloning Kit (Vazyme). The *VaPYL4* gene driven by the 35S promoter was located at 5’ upstream region of *EGFP* gene in the 35S::*VaPYL4*-*EGFP* vector (Additional file 1: Figure S2). The developed construct was introduced into the *Agrobacterium* strain GV3101, which was used for infiltration of *Nicotiana benthamiana* leaves. The fluorescence was detected 3 days after infiltration using Leica TCS SP8 confocal laser scanning microscopy. The histone H2B-mCherry was used as an indicator of nucleus as previously described [33].

### Plant transformation

To develop the vector for gene overexpression in grape callus and *Arabidopsis*, the *VaPYL4* gene was amplified from the pLB vector using the primers PYL4-P277-F and PYL4-P277-R and ligated into the *Eco*RI-digested pSAK277-EGFP vector via HR. The overexpression vector was introduced into the *Agrobacterium* EHA105 and GV3101 for the transformation of grape callus and *Arabidopsis*, respectively. The transformation of grape callus was conducted by using petioles of *V. amurensis* plants as the explants according to the protocol described previously [53]. The petioles were placed on Gamborg medium supplemented with 50 mg L^−1^ kanamycin after co-culture with *Agrobacterium* cells and sub-cultured monthly until kanamycin-resistant calli were developed. EGFP fluorescence was detected using CCD camera (Tanon 5200) to select transgenic calli rapidly. Then the calli were identified by PCR using *NPT II*-specific primers (Additional file 1: Table S1). The expression of *VaPYL4* in the induced calli was further confirmed by qPCR. *Arabidopsis* transformation was performed using the floral-dip method [34]. Transgenic plants were screened on 1/2 MS medium with 50 mg L^−1^ kanamycin. The T_3_ homozygous transgenic lines were used for the treatments.

### Targeted mutagenesis of *VaPYL4*

To knock out *VaPYL4* gene in grape, two different targets were designed to target the exon of *VaPYL4* using the targetDesign tool of CRISPR-GE (http://skl.scau.edu.cn/targetdesign/). The designed sgRNAs were ligated to grape VvU6.1 and VvU3.1 promoter [54] to develop sgRNA expression cassettes. Then the sgRNA expression cassettes were inserted into pCACRISPR/Cas9 vector [55] through *Eco*RI and *Hin*dIII sites via HR. The construction of the CRISPR vector was carried out as previously described [55]. The well-constructed CRISPR vector was introduced into grape callus by *Agrobacterium*-mediated transformation [53]. To detect targeted mutagenesis, the induced calli were sampled for genomic DNA extraction, and the DNA fragment containing the target sequence was amplified from genomic DNA. The PCR amplicons were cloned into the pLB vector, and a number of 20 clones were analyzed by Sanger sequencing for each callus.

### qPCR assay

For qPCR assay, the cDNA was synthesized from total RNA using the HiScript II Q RT SuperMix for qPCR Kit (Vazyme). The qPCR was performed using AceQ qPCR SYBR Green Master Mix (Vazyme) with the CFX Manager system (BioRad). The reactions were carried out as described by Ren et al. [56]. Grape *Actin 1* and *GAPDH* [33] and *Arabidopsis Actin 2/8* were used as internal controls. Gene expression relative to internal controls was determined using 2^−∆∆CT^ method [57]. Significant differences were determined by Student’s *t-*test.

### Plant phenotyping

The plant with at least one flower (Additional file 1: Figure S8) was considered to be at the flowering stage, and the growing time for development of the first flower was recorded as the flowering time for each plant. For the measurement of fresh weight of seedlings, each plant was measured without roots, considering that the soil cannot totally be removed from the roots. To measure the weight of siliques, all the siliques from the same genotype were pooled and a number of 10 siliques were collected as a replicate. The fresh weight was determined at 50 d post-germination.

### Measurement of LTEs and Physiological assays

The cold tolerance of grape calli was evaluated by its LTEs, which were measured using the Keithley Multimeter Data Acquisition System (model 2700-DAQ-40) combined with a programmable freezer and a Tenney Environmental Test Chamber (model T2C, Thermal Product Solutions) as previously described [36]. At least 5 biological replicates were used for the measurement. Electrolyte leakage of *Arabidopsis* plants was analyzed using the method described by Li et al. [57]. Electrolyte leakage assay was repeated three times. To measure the MDA content and POD activity, the plants were ground into powder with liquid nitrogen, and 0.1 g of powder was used for the measurement. The MDA level and POD activity were determined using MDA and POD isolation kits (Solarbio) following the manufacturer’s instructions. Five biological and three technical replicates were conducted.

## Supplementary Information

**Additional file1.**

## Data Availability

The transcriptome date used in this study are available in NCBI Gene Expression Omnibus (GEO) database under the accession number of GSE166247. The data generated or used in this study are included in the manuscript and supplementary materials.

## Abbreviations

ABA: Abscisic acid
CDS: Coding sequence
COR15A: Cold responsive 15A
CRISPR/Cas9: Clustered regulatory interspaced short palindromic repeats/CRISPR-associated protein 9
EGFP: Enhanced green fluorescent protein
EV: Empty vector
FW: Fresh weight
HR: Homologous recombination
KIN2: Kinase 2
KO: Knockout
LTEs: Low temperature exotherms
MDA: Malondialdehyde
OE: Overexpression
POD: Peroxidase
PYL: Pyrabactin resistance-like proteins
qPCR: Quantitative real-time PCR
RD29A: Responsive to desiccation 29A
WT: Wild type
