# Supplementary material for: Overexpression of grape ABA receptor gene VaPYL4 enhances tolerance to multiple abiotic stresses in Arabidopsis
Source: BMC Plant Biol. 2022 Jun 2;22:271. doi: 10.1186/s12870-022-03663-0 (PMC9161562; doi:10.1186/s12870-022-03663-0)
Supplement: Supplementary file 1 — Additional file1. [file 12870_2022_3663_MOESM1_ESM.pdf]

Supplementary Figures and Tables

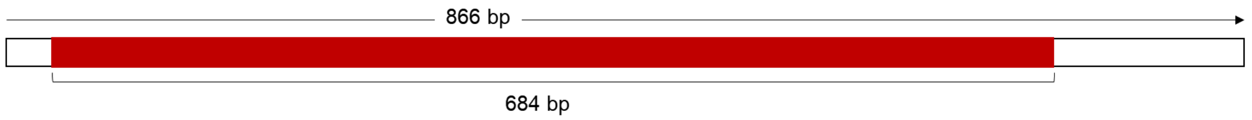

Fig.S1 Genomic structure of the *PYL4* gene. The exon is showed in red and the upstream and downstream untranslated regions (UTRs) are shown in white.

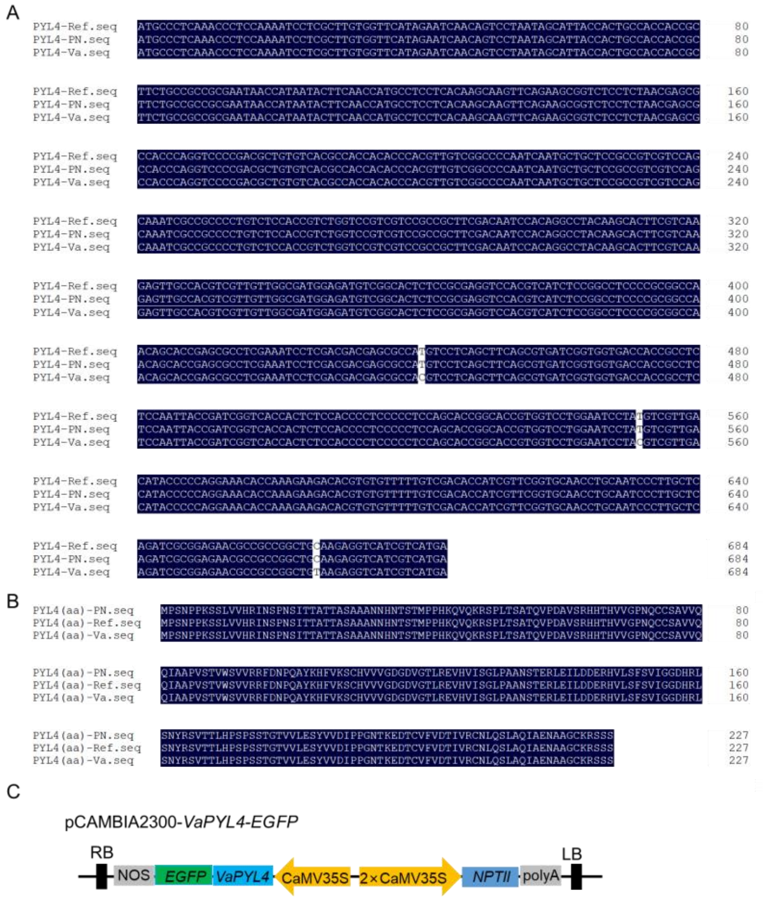

Fig.S2 The amplified sequences of *PYL4* genes. **A.** Alignment of *PYL4* genes amplified from *V. vinifera* cv. Pinot Noir and *V. amurensis*. The *PYL4* sequence from the reference genome (PN40024) was used as the reference sequence. **B.** Corresponding amino acid sequences encoded by the genes shown in A. **C.** Schematic diagram of T-DNA region of the pCAMBIA2300-VaPYL4-EGFP vector for subcellular localization assay. NOS, terminator of nopaline synthase gene; EGFP, enhanced green fluorescent protein gene, CaMV35S, cauliflower mosaic virus 35S promoter; NPTII, neomycin phosphotransferase gene; polyA, cauliflower mosaic virus polyadenylation signal; RB, right border; LB, left border.

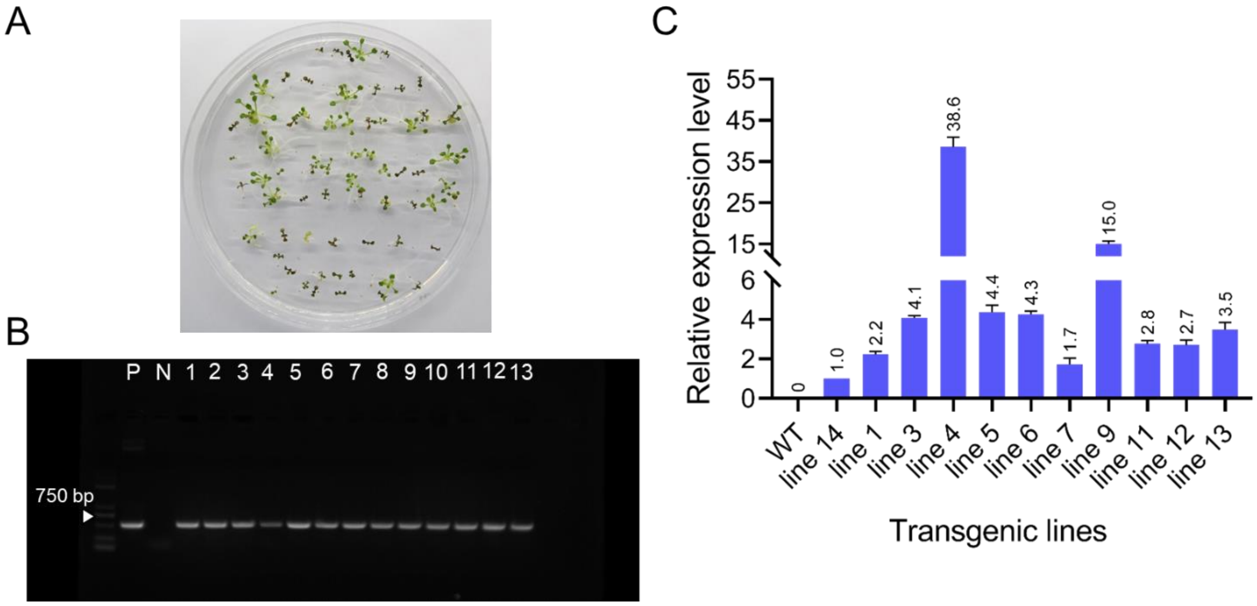

Fig.S3 Identification of *VaPYL4*-overexpressing *Arabidopsis* lines. **A.** *Arabidopsis* seeds germinated on 1/2 MS medium supplemented with 50 mg/L kanamycin. **B.** PCR identification of *NPT II* gene in kanamycin-resistant plants shown in A. The plasmid and wile-type plant were used as positive (P) and negative (N) control, respectively. **C.** Expression level of *VaPYL4* gene in different transgenic lines.

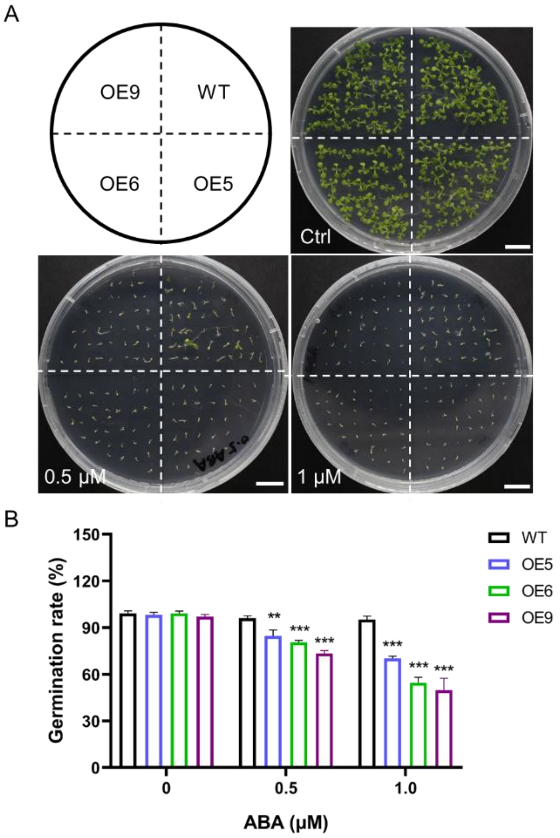

Fig.S4 Germination of wild-type (WT) and *VaPYL4*-overexpressing (OE) seeds with or without exogenous ABA. **A.** Seeds germination on 1/2 MS medium. After imbibition in water for 2 d at 4 °C, the seeds of WT and OE were sown on 1/2 MS medium. The emergence of radicle was measured as germination, and the germination rates were calculated at 7 d after sowing. Scale bars correspond to 1 cm. **B.** Germination rates of *Arabidopsis* seeds on 1/2 MS medium shown in A. Data are collected from three replicates and shown as averages of around 120 seeds. Date are presented as means ± SD. $^{**}P < 0.01$, $^{***}P < 0.001$.

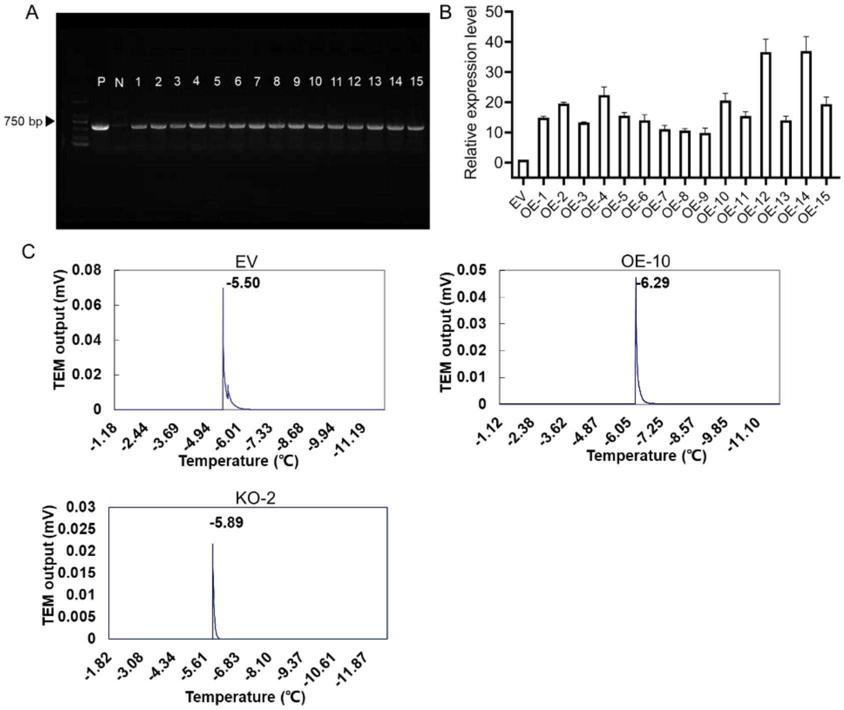

Fig.S5 Identification of transgenic grape calli and low-temperature exotherms (LTEs) measurement of grape calli. **A.** PCR identification of T-DNA insertions using *NPT II*-specific primers. The plasmid of overexpression vector and wild-type callus were used as positive (P) and negative (N) controls, respectively. Lanes 1-15, independent grape calli. **B.** Expression levels of VaPYL4 in transgenic grape calli. The expression of VaPYL4 in OE (overexpression) lines relative to EV (empty vector control) was determined by qPCR. Data are collected from three biological replicates. **C.** LTEs measurement of grape calli. The calli were put on thermoelectric modules (TEMs), and exotherms were identified manually from a plot of thermistor output (x-axis) versus loaded TEM output minus the empty TEMs output (y-axis).

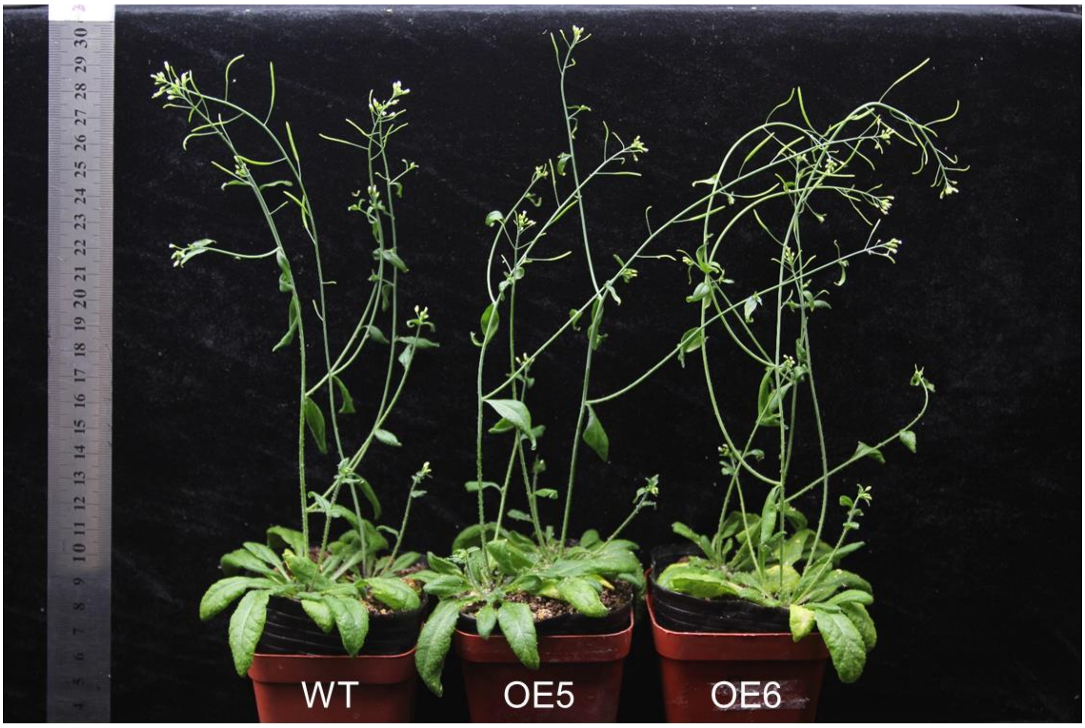

Fig.S6 *Arabidopsis* plants grown under normal conditions. This photo showing the control group of multi-stress treatment was taken at the end of the treatment (46 days post-germination).

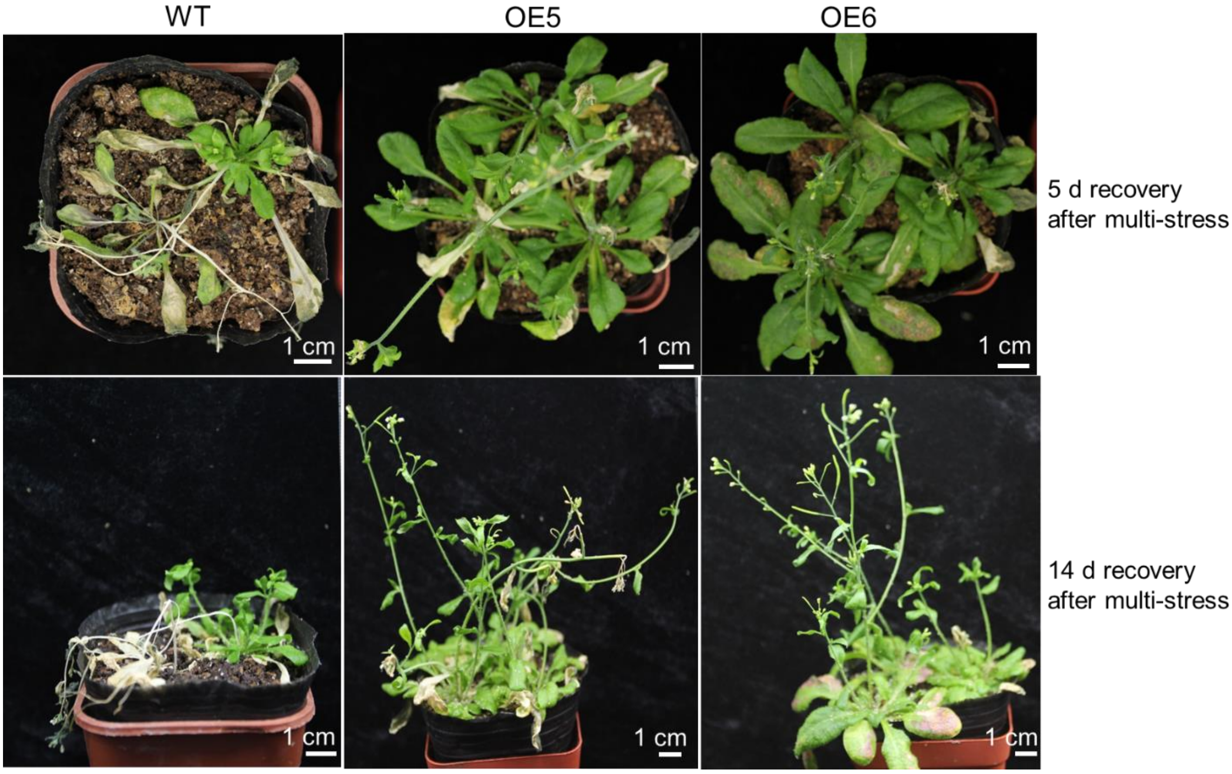

Fig.S7 Phenotypes of wild-type (WT) and transgenic (OE5 and OE6) plants after multi-stress treatment. The treatment was performed as shown in Fig. 7A.

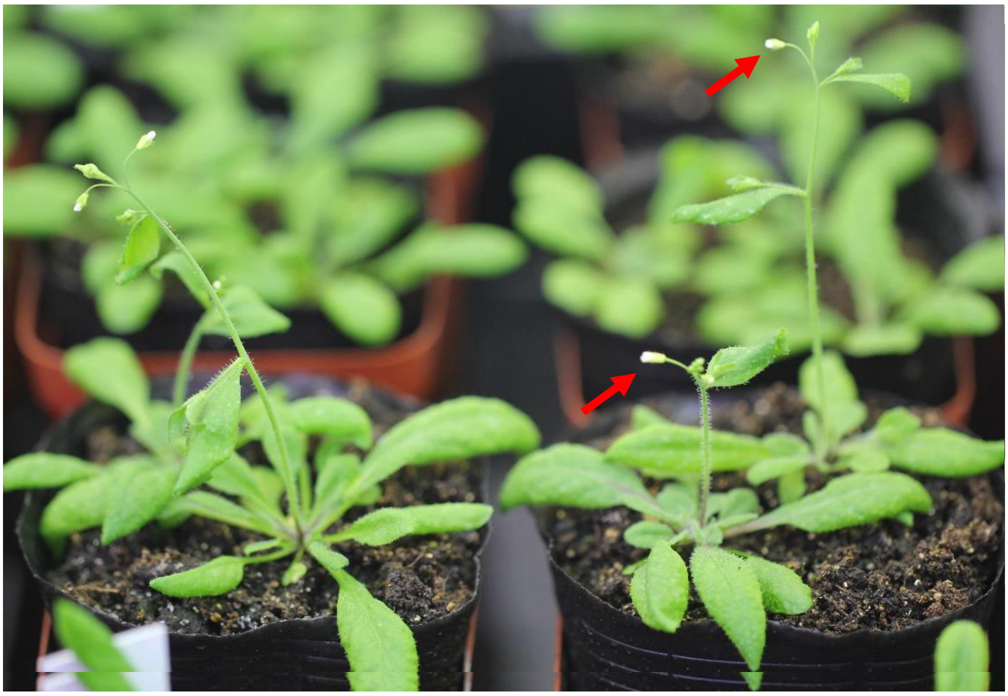

Fig.S8 *Arabidopsis* plants at the flowering stage. The plants with at least one flower (indicated by red arrows) was regarded to be at the flowering stage.

Table S1. Primers used in this study.

**Primer name**	**Primer sequence (5'-3')**	**Experiment**
PYL4-PCR-F	AATGCCCTCAAACCCTCCAAAAT	Gene cloning and construction of overexpression vectors
PYL4-PCR-R	TCTGCTCATGACGATGACCTC	
PYL4-2300-F	ACGGGGGACGAGCTCGGTACCATGCCCTCAAACCCTCCAAAAT	
PYL4-2300-R	TCTAGAGGATCCCCGGGTACCTGACGATGACCTCTTGCA	
PYL-P277-F	TCGACACTAGTGGATCCAAAATGCCCTCAAACCCTCCAAAAT	
PYL-P277-R	AGCTTCCCGGGGGTACCGGGTGACGATGACCTCTTGCA	
NPT II-PCR-F	GGCTATGACTGGGCACAACA	PCR identification
NPT II-PCR-R	TGAATCCAGAAAAGCGGCCA	
VvU6.1-gR1-F1	CAGGAAACAGCTATGACATGTTGCCTCTGGAAAATCC	Construction of CRISPR expression vector
VvU6.1-gR1-R2	CCCTATAGTGAGTCGTATTACGGTTCACTAAACCAGCTCT	
VvU3.1-gR2-F1	TAATACGACTCACTATAGGGAGTACTTTCATAGGAATAG	
VvU3.1-gR2-R2	GTAAAACGACGGCCAGTGCCGGTCGACTCTAGCGGATCTG	
VvU6.1-PYLg1-R1	CGCTTGTGGTTCATAGAATCAAGCTCTAAGCGTTTGC	
VvU6.1-PYLg1-F2	GATTCTATGAACCACAAGCGGTTTTAGAGCTAGAAATAGC	
VvU3.1-PYLg2-R1	CTTCTGCCGCCGCGAATAACTGGCATGTGAATCTCCTATC	
VvU3.1-PYLg2-F2	AGTTATTCGCGGCGGCAGAAGGTTTTAGAGCTAGAAATAGC	
PYL4-KO-PCR-F	AAAGCGTCCTCAATGTGGTGA	Identification of targeted mutagenesis
PYL4-KO-PCR-R	TAATTGGAGAGGCGGTGGTCA	
SOD-qPCR-F	TCCATTCGCACTGGATGCTT	qPCR
SOD-qPCR-R	AGGTTGTCCACGTAAGCTCTG	
LOX2-qPCR-F	AGACTGACCAGCGGATTACG	

LOX2-qPCR-R	TCAGGCATCTCAAACTCGCA	
RD29A-qPCR-F	TTCCACCAGGGACAAAGGTG	
RD29A-qPCR-R	TGCATCGTGTCCGTAAGAGG	
COR15A-qPCR-F	ATCCACTTAGCCATTCCAAGCA	
COR15A-qPCR-R	TGTGACGGGAGATGAGGTGA	
COR15B-qPCR-F	AACCACAACTTGATGGCCGA	
COR15B-qPCR-R	AGAAGAGTTTTCGTTGGTTCGT	
KIN2-qPCR-F	GCGATCCGAGTCAACTTTGG	
KIN2-qPCR-R	CAACAAGTACGATGAGTACGAGA	
PYL4-qPCR-F	GTTTTTGTCGACACCATCGTT	
PYL4-qPCR-R	TCATGACGATGACCTCTTGC	
Actin2/8-qPCR-F	GGTAACATTGTGCTCAGTGGTGG	
Actin2/8-qPCR-R	AACGACCTTAATCTTCATGCTGC	
